# Stable socioeconomic inequalities in ischaemic heart disease mortality during the economic crisis: a time trend analysis in 2 Spanish settings

**DOI:** 10.1186/s13690-019-0339-z

**Published:** 2019-03-15

**Authors:** Xavier Bartoll, Mercè Gotsens, Marc Marí-Dell’Olmo, Laia Palència, Montse Calvo, Santiago Esnaola, Carme Borrell

**Affiliations:** 1 Agència de Salut Pública de Barcelona, Plaça Lesseps 1, 08023 Barcelona, Spain; 2 CIBER Epidemiología y Salud Pública (CIBERESP), Madrid, Spain; 3 Institut d’Investigació Biomèdica (IIB Sant Pau), Barcelona, Spain; 4 Universitat Pompeu Fabra, Barcelona, Spain; 5 Departamento de Salud, Gobierno del País Vasco, Bilbao, Spain

**Keywords:** Ischaemic heart disease mortality, Socioeconomic inequalities, Economic, Crisis, Spain

## Abstract

**Background:**

Prior studies have identified a decrease in ischaemic heart disease mortality during the recent economic recession. The Spanish population was severely affected by the Great Recession, however, there is little evidence on its effects on socioeconomic inequalities in ischaemic heart disease mortality. This study examines trends in socioeconomic inequalities in mortality due to ischaemic heart disease (IHD).

**Methods:**

We used linked census records with mortality registers available from the Basque Country and Barcelona city for population above 25 years, between 2001 and 04, the accelerated economic growth period of 2005–08, and 2009–12, with the last period coinciding with the Great Recession. Applying Poisson models, we calculated relative and absolute indexes of inequalities by education level for each period, age group, gender, and site.

**Results:**

We found moderate age-adjusted inequalities in IHD with a gradient of increasing rates through less educational level, but no significant evidence of increasing trends in socioeconomic inequalities in IHD mortality, rather an inverted U-shape time trend in some groups below 75 years in relative inequalities. Absolute inequalities decrease in the last period except for women from 50 to 64 years.

**Conclusions:**

This study shows that the economic crisis has not increased socioeconomic inequalities in IHD mortality in two geographical settings in Spain.

## Background

An influential study in the late 70’s found that economic recessions were related to increased cardiovascular mortality [1]. This result was soon challenged [2] by new evidence based on more sound panel methodology showing just the opposite, that the increased unemployment was associated with a decline in cardiovascular mortality [3]. The beginning of the Great Recession in 2008 has brought renewed interest in analysing the effect of economic cycles on mortality. However, there is little evidence on the effects of the economic cycles on specific and all-cause mortality inequalities by socioeconomic level. It is well reported that lower socioeconomic groups have higher mortality rates across European countries [4, 5]. One study for the period 1980–1990 in USA reported that men with less education experienced higher all cause-mortality with increasing unemployment [6]. While in Nordic countries and Japan characterized with universal health care, the mortality in cardiovascular disease of lower socioeconomic group decreased [7–9]. Another study for Northern Ireland found no significant increase in ischaemic heart disease mortality among the working population after the economic crisis in 2008, but an increase for retired workers [10]. For Spain, decreases in all-cause mortality and cardiovascular disease during the Great Recession were found larger for lower socioeconomic groups (proxied by household floor space and car ownership) [11].

After the mild crisis of 2001, the Spanish economy grew at a constant rate for the next years until the fourth quarter of 2008 to enter in negative growth rates. Since then, the economic recession has severely affected the Spanish population, with a rapid rise in unemployment, work precariousness and vulnerable population increasing socioeconomic inequalities [12]. Moreover, as in the European context, the austerity measures imposed in Spain may have negatively affected health and health services [13, 14].

To analyse trends in socioeconomic inequalities of ischaemic heart disease (IHD) mortality we compared two distinct settings in Spain, for which data were available: Barcelona city and The Basque Country. Both areas have similar populations and are in the north of Spain. The Basque Country had the lowest unemployment rate (rose from 11.6% in 2001 to 14.9% in 2012) and poverty rates in Spain (ranged from 5 to 10% even during the crisis period) and ranked first in health spending per capita (reached the peak of 1971€ in 2009). Conversely in Barcelona, the unemployment rate rose from 10.5% to a 18.7% and poverty rate ranged from 15.7 to 18.3% in the same period, own estimation of health spending per capita for Barcelona area are lower (reached the peak of 1398€ in 2009). The figures in Barcelona were closer to the Spanish average than those of The Basque Country [15–17].

The objective of this paper is to analyse trends in socioeconomic inequalities of IHD mortality before (periods 2001–04 and 2005–08) and during the Great Recession (2009–12) according to education level.

## Methods

### Design, study population and sources of information

This study uses individual-level data from the Basque Country and Barcelona city, two settings where the information of the mortality register linked with the census is available in Spain.

The Basque Country population was 2,082,587 at the end of 2001 and 2,179,815 at the end of 2011. Barcelona is the second largest city in Spain with 1,505,325 inhabitants in 2001 and 1,620,943 in 2012. We considered at-risk population to be all residents older than 25 years between 2001 and 2012. We choose 25 years as cut-off point because university studies are finished at this age.

Barcelona and the Basque country have two different register information. In Barcelona it is possible to link cases of the people who died, while in Basque Country uses a follow up linkage. For Barcelona, we only included IHD deaths in the mortality register that occurred among residents between 2001 and 2012, and for whom complete educational level information was available. Data on subjects’ educational level was obtained by record linkage between the mortality register and Barcelona’s municipal census. Information on the at-risk population, including age and sex and was obtained from the municipal census. Due to a lack of linkage, we excluded 401 IHD deaths for men (4.4%) and 332 for women (4.7%).

For the Basque Country, we used a population-based census-linked longitudinal mortality study. Individual census records from the 2001 and 2006 population censuses were linked with the mortality registry. Individuals from the 2001 census were followed up from November 2001 (date of the 2001 Census) to October 2006, and those from the 2006 census were followed up from November 2006 to December 2012. Deaths were weighted by the inverse of the proportion of linked cases. The exact number of person-years of follow-up was calculated by subtracting the date of the corresponding census from the date of death (for deceased persons) or the date of the end of follow up. The percentages of male and female IHD deaths for whom no information on educational level was available due to a lack of linkage were 3.3 and 4.8% for the 2001–2004 period, 2.8 and 3.3% for the 2005–2008 period, and 4.8 and 2.7% for the 2009–2012 period, respectively.

### Variables and indicators analysed

We analysed deaths due to IHD included International Classification of Diseases 10th (ICD-10) codes of the underlying cause of death: from angina pectoris (I20) to chronic ischaemic heart disease (I25). Our main independent variable was the highest level of education attained. Educational level was categorised in five groups: (a) no formal education or pre-primary education (0–4 years of schooling), (b) primary education (5–6 years of schooling); (c) lower secondary education (7–9 years of schooling); (d) upper secondary education (10–14 years of studies), and (e) university education (≥15 years of schooling). Although education level from census records is not always an up-to-date register during the life-course, it is attained relatively early in life and it tends to be stable over the adult life span, moreover it has been extensively used in mortality analysis as a proxy for socioeconomic position in most of the aforementioned literature. Other independent variables were sex, age, and year of death. Age of death was grouped in five categories: 25–49 years, 50–64 years, 65–74 years, ≥75 years, and an all-ages group. Year of death was grouped into two periods before the beginning of the economic recession (2001–04 and 2005–08) and one period after the beginning of the recession (2009–12). Although the economic recession actually started during the 4th term of 2008, unemployment levels did not start to rise noticeably until 2009.

### Data analysis

All analyses were carried out separately for each region (Barcelona and Basque Country), for men and women, for the four age groups and all-ages group, and for the three-time periods. We fit Poisson regression models to compute the Relative Index of inequality (RII) and its 95% confidence interval (95% CI) for age-adjusted educational level specific mortality. The dependent variable was the logarithm of the mortality rate, and the independent variables were educational level as a quantitative variable (with five values between 0 and 1, based on the midpoint of the educational level group’s position in the cumulative educational level distribution of the population). The RII value can therefore be interpreted as the ratio of mortality rates between the two extremes of the educational spectrum (in our case, the lowest educational level versus the highest, taking into account all educational levels). We also fit models with the 3 periods together where the independent variables were age group, period, educational level and the interaction between period and educational level, to determine if the RII changed over time.

Moreover, we estimated the age-adjusted Slope Index of Inequality (SII). To estimate SII, simply adjusting for age may not be enough to obtain a truly comparable measure, so we used a novel approach [18]. First, different SII were obtained for each age-group with an additive Poisson model. Second, we obtained a single SII performing a weighted sum of these SII. The weights correspond to the relative sizes of the age groups in a reference population (in our case both areas Barcelona and Basque Country). The regression models are adjusted by the 5-year variable. Poisson models were corrected for overdispersion where necessary. Due to the increase of immigration to Spain in the last two decades, we have replicated all the analysis stratifying by country of birth, as the results were not significantly different, we have only reported the results for the overall population.

## Results

Table 1 shows descriptive of the number and rate of crude IHD deaths, according to level of education, age-group, sex and period at the two sites. In Barcelona we observe a decreasing trend among those at the extreme of the education level, no education and primary as well as among those in university level, while for those in lower secondary level from 50 to 64 years that increased across 2001–2012 and those above 75 years that increased in the last period. For women, rates decrease for most groups except for those in lower secondary from 50 to 64 years that increases in the last period, and university level from 65 to 74 years. For men in Basque Country, IHD deaths rates decrease for most groups, except for those in university from 50 to 64 years and lower secondary above 75 years. For women, it decreases in the last period, except for women from 50 to 64 years that increases except for those in primary education level.

**Table 1:** Number and crude rate of deaths (per 100.00 population) due to ischaemic heart disease for Barcelona and the Basque Country by age group, 2001–2012

Region/Age groups (years)	Education level	Men						Women					
		2001–2004		2005–2008		2009–2012		2001–2004		2005–2008		2009–2012	
		n	rate	n	rate	n	rate	n	rate	n	rate	n	rate
Barcelona													
25–49	No education	5	22.0	2	9.3	1	3.4	2	10.5	1	7.0	0	0.0
	Primary	36	18.9	26	11.3	19	8.7	3	1.9	9	5.3	2	1.3
	Lower secondary	31	12.1	30	11.4	27	10.3	1	0.4	2	0.8	4	1.9
	Upper secondary	31	7.3	27	6.1	25	6.2	4	1.0	4	0.9	4	1.0
	University	16	5.9	12	3.8	12	3.1	4	1.2	1	0.3	1	0.2
50–64	No education	58	112.6	38	118.1	15	80.4	18	21.5	8	14.3	3	9.4
	Primary	152	99.7	131	94.1	81	68.1	28	13.7	20	10.6	15	9.4
	Lower secondary	61	65.7	74	74.1	85	80.0	6	4.9	8	5.8	13	8.8
	Upper secondary	80	77.0	62	50.1	88	59.7	11	12.8	10	9.4	9	6.8
	University	75	65.1	69	51.7	51	34.3	3	3.3	9	7.4	6	3.8
65–74	No education	207	290.9	143	271.1	76	218.7	125	98.0	78	82.8	47	73.2
	Primary	295	284.9	217	235.6	167	197.5	109	70.2	68	49.4	33	26.1
	Lower secondary	84	212.4	91	225.6	90	202.4	31	61.3	20	36.8	20	31.8
	Upper secondary	127	294.2	86	197.2	95	203.0	17	47.1	15	39.8	9	20.8
	University	107	246.2	75	164.6	71	127.9	13	46.9	7	22.9	12	30.0
> =75	No education	600	991.6	498	810.9	383	636.8	1154	677.4	839	506.9	720	461.8
	Primary	717	974.6	651	790.6	642	714.8	828	509.6	804	448.0	714	372.2
	Lower secondary	204	850.6	186	659.7	223	663.6	142	376.2	148	320.2	173	308.2
	Upper secondary	268	952.4	224	690.4	269	717.4	123	448.8	107	312.7	124	304.8
	University	259	928.5	268	801.3	297	739.0	107	496.7	83	321.6	101	320.5
Basque Country													
25–49	No education	2	22.8	6	50.0	1	8.0	0	0.0	0	0.0	0	0.0
	Primary	89	22.9	89	19.8	80	20.5	13	3.5	14	3.6	9	3.0
	Lower secondary	22	48.1	10	19.9	2	4.5	7	13.3	2	3.8	2	4.9
	Upper secondary	46	9.2	62	8.8	89	12.2	4	1.0	14	2.5	6	1.0
	University	27	7.0	21	4.2	17	3.5	4	0.9	5	0.8	5	0.8
50–64	No education	28	112.5	37	161.4	21	132.2	7	20.4	5	15.7	6	28.2
	Primary	285	91.6	300	80.6	266	78.1	63	15.7	44	9.2	37	8.7
	Lower secondary	31	61.9	65	79.6	59	66.3	2	3.2	8	7.5	11	9.2
	Upper secondary	91	75.7	136	69.2	146	58.5	2	3.3	6	5.1	11	6.2
	University	62	69.9	77	57.5	96	59.0	5	9.4	8	8.2	11	7.6
65–74	No education	91	250.9	93	262.8	32	127.0	41	77.5	40	76.4	20	52.9
	Primary	504	244.7	450	192.3	347	159.3	167	62.7	146	47.0	102	34.6
	Lower secondary	26	206.1	39	179.9	44	141.5	10	63.8	12	40.2	11	24.4
	Upper secondary	66	227.5	86	187.5	77	123.4	3	21.3	8	34.5	11	32.4
	University	55	203.0	59	155.4	68	145.2	7	50.1	6	29.8	3	11.3
> =75	No education	310	993.7	308	752.4	297	710.3	423	586.0	509	556.1	451	498.1
	Primary	1081	836.3	1266	657.7	1337	598.7	1100	459.3	1368	394.6	1268	320.0
	Lower secondary	35	633.6	57	514.3	112	667.0	38	431.1	67	333.7	96	306.8
	Upper secondary	88	758.0	140	619.8	176	532.4	27	302.9	45	265.3	63	260.5
	University	91	647.5	148	641.4	154	514.3	52	460.8	58	325.1	54	238.2

Regarding standardized rate deaths of IHD in all ages group in Table 2, we observe a decrease through the period 2001–2012 in Barcelona among all education level men and women except for those men in upper secondary level that increases in the last period. For instance, men with no education rates ranges from 221.5 per 100.00 inhabitants in 2001–2004 to a 140.0 in 2008–2012, those in university from 197.2 to a 140.7 in the same period. For women with no education, IHD rates range from a 106.9 in 2001–2004 to a 66.0 in 2008–2012, and for those in university level from 76.5 to a 47.7 in the same period. In Basque Country, standardized death rates of IHD decreases among men and women through the period. For instance, men with no education rates range from 196.3 in 2001–2004 to a 130.0 in 2008–2012, those in university from 135.6 to 107.1 in the same period. For women with no education, IHD rates range from 75.4 to 65.0 and among those with university degree from 62.3 to 29.7.

**Table 2:** Number and age-standardized rate of deaths (per 100.00 population) due to ischaemic heart disease, Barcelona and the Basque Country, 2001–2012

Region/Age groups (all ages)	Education level	Men						Women					
		2001–2004		2005–2008		2009–2012		2001–2004		2005–2008		2009–2012	
		n	rate	n	rate	n	rate	n	rate	n	rate	n	rate
Barcelona	No education	870	221.5	681	185.8	475	140.0	1299	106.9	926	79.0	770	66.0
	Primary	1200	222.1	1025	181.0	909	151.8	968	86.3	901	70.9	764	51.1
	Lower secondary	380	185.3	381	156.9	425	153.3	180	67.2	178	53.9	210	50.3
	Upper secondary	506	209.4	399	150.2	477	156.1	155	81.1	136	54.5	146	46.3
	University	457	197.2	424	160.2	431	140.7	127	76.5	100	49.1	120	47.7
Basque Country	No education	431	196.3	444	185.6	351	130.0	471	75.4	554	70.2	477	65.0
	Primary	1.959	179.8	2.105	142.5	2.030	124.9	1.343	68.2	1.572	55.7	1.416	42.6
	Lower secondary	114	149.7	171	125.7	217	118.9	57	69.4	89	48.0	120	44.5
	Upper secondary	291	165.2	424	131.9	488	118.1	36	43.5	73	42.6	91	35.6
	University	235	135.6	305	125.0	335	107.1	68	62.3	77	43.0	73	29.7

**Fig. 1:**
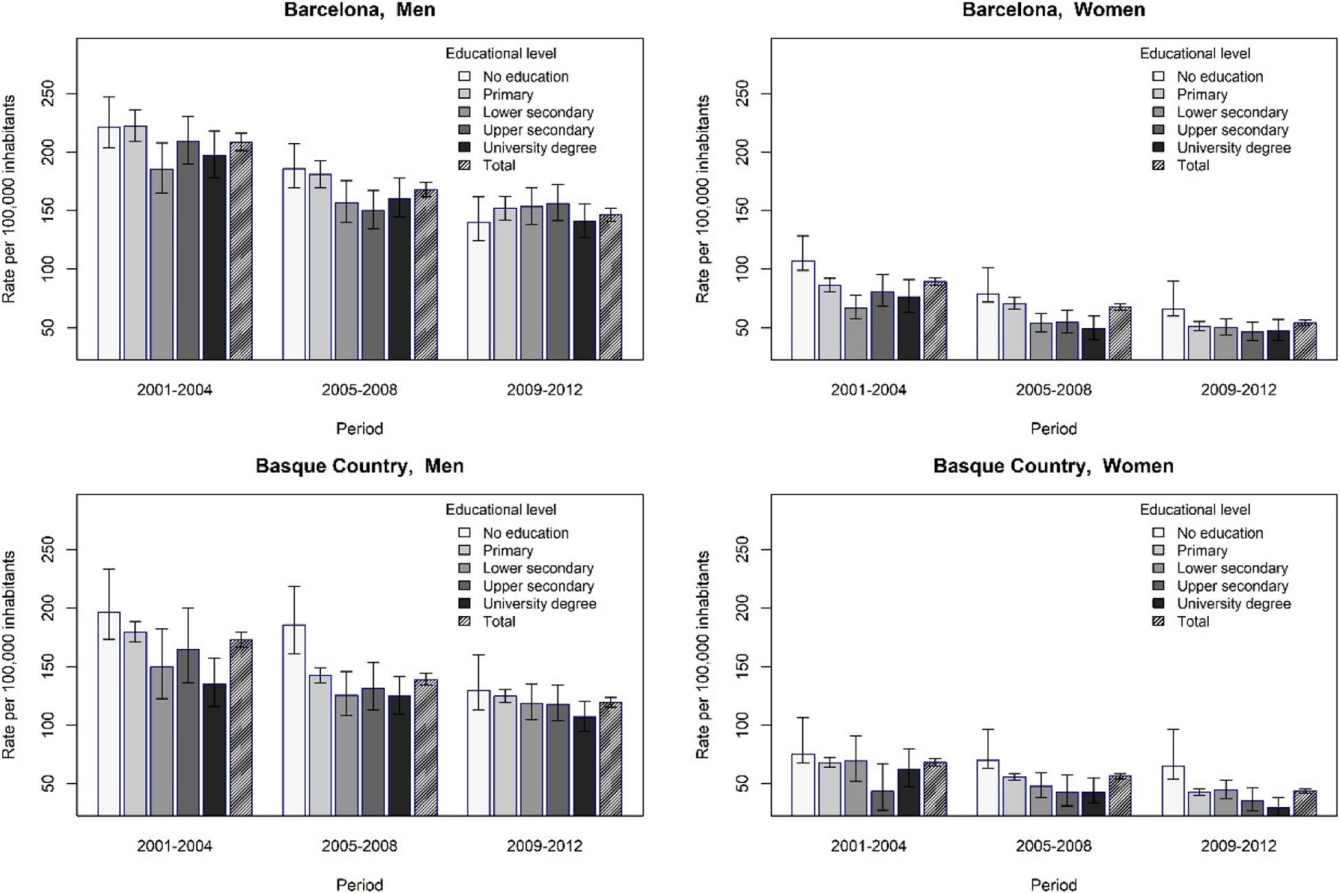
Ischaemic heart disease age-standardized mortality rates and 95% confidence intervals. Barcelona and the Basque Country. 2001–2012

Figure 1 shows a moderate inequality gradient in mortality by education level for the all-ages groups, with the rate increasing with decreasing educational level. The pattern of inequality is quite stable across time. IHD death rates for men in no education level decrease more than those of high educational level in both settings while for women they are more stable.

To better analyse the evolution of these inequalities by education level across periods, Table 3 shows the relative index of inequality (RII) and the slope index of inequality (SII), age group, sex, and study period. Most RIIs were greater than 1, which means that those in lower educational level have higher risk of death from IHD than higher educational level ones. The most consistent finding is that there was no significant change in RII between study periods as confidence intervals overlap and the interaction between period and educational level is not significant (interaction *p*-values not shown). However, for men and women in Barcelona the RII appears to show an inverted U-shape trend, that is, it is lower in the first and last periods but higher in the middle one (except for women from 50 to 64 years that follows a U-shape trend and for both sexes over 74 years). Nonetheless the time variations of the RII are of minimum magnitude. For instance, the RII for men in all ages group during 2001–2004 is 1.2 (95%CI:1.0–1.4), then increases to 1.3 (95%CI: 1.1–1.6) and finally decreases to 1.1 (95%CI:0.9–1.3) in 2009–2012. For women in Barcelona the figures are, first 1.7 (95CI%:1.3–2.1), then increases to 1.8 (95CI%:1.5–2.3) and finally decreases to 1.5 (95%CI:1.2–1.9). This pattern is common across age groups for men and for women. The RII for men in the Basque Country also follows the inverted U-shape across periods for each age group (except for those over 74 years), while for women tend to increase through time. For instance, in all age group the RII varies from 1.5 (95%CI:1.1–1.9) in 2001–2004 to a 1.9 (95%CI:1.5–2.4) in 2005–2008 and finally to a 2.1 (95%CI:1.5–2.8). This pattern repeats for each age group (except for those from 50 to 64 years that follows a U-shape trend). As said, none of these variations are significant so we must conclude stability in relative inequalities.

**Table 3:** Relative and absolute inequality measures of ischaemic heart disease mortality for Barcelona and the Basque Country, 2001–2012

Age group (years)		25–49		50–64		65–74		> = 75		All ages	
Region/Sex	Period	RII	95% CI	RII	95% CI	RII	95% CI	RII	95% CI	RII	95% CI
Barcelona											
Men	2001–04	4.2	(1.8–10.0)	1.6	(1.2–2.3)	1.1	(0.9–1.5)	1.1	(0.9–1.2)	1.2	(1.0–1.4)
	2005–08	4.9	(2.4–10.4)	2.3	(1.3–4.2)	1.6	(1.2–2.2)	1.1	(0.9–1.3)	1.3	(1.1–1.6)
	2009–12	4.5	(1.9–10.1)	2.0	(1.2–3.5)	1.4	(0.8–2.5)	0.8	(0.7–0.9)	1.1	(0.9–1.3)
Women	2001–04	1.8	(0.2–15.5)	3.7	(1.0–13.4)	2.2	(1.5–3.4)	1.5	(1.2–1.9)	1.7	(1.3–2.1)
	2005–08	33.6	(3.5–322.2)	1.6	(0.6–4.2)	3.4	(1.7–6.6)	1.4	(1.2–1.7)	1.8	(1.5–2.3)
	2009–12	7.9	(0.7–84.7)	2.0	(0.6–6.5)	3.2	(1.3–7.8)	1.4	(1.1–1.7)	1.5	(1.2–1.9)
Basque Country											
Men	2001–04	3.3	(1.8–5.7)	1.4	(1.0–2.0)	1.2	(0.9–1.6)	1.4	(1.1–1.7)	1.5	(1.3–1.8)
	2005–08	5.3	(2.7–10.3)	1.5	(1.0–2.3)	1.4	(1.1–1.9)	1.2	(0.9–1.4)	1.4	(1.2–1.7)
	2009–12	4.1	(2.0–8.2)	1.5	(0.9–2.2)	1.1	(0.7–1.6)	1.2	(0.9–1.5)	1.3	(1.1–1.6)
Women	2001–04	3.2	(0.7–14.4)	4.3	(1.2–15.1)	1.6	(0.9–2.9)	1.2	(0.9–1.5)	1.5	(1.1–1.9)
	2005–08	4.3	(1.2–15.5)	1.4	(0.5–3.7)	2.1	(1.2–3.9)	1.6	(1.3–1.9)	1.9	(1.5–2.4)
	2009–12	6.7	(1.2–38.7)	1.7	(0.4–6.8)	2.4	(1.2–4.8)	1.7	(1.4–2.1)	2.1	(1.5–2.8)
Region/Sex	Period	SII	95% CI	SII	95% CI	SII	95% CI	SII	95% CI	SII	95% CI
Barcelona											
Men	2001–04	14.9	(6.6–23.4)	39.7	(12.1–67.4)	38.7	(−32.4–109.9)	58.5	(−106.4–223.5)	29.0	(4.4–53.6)
	2005–08	13.1	(7.6–18.5)	58.6	(27.0–90.1)	110.9	(49.4–172.5)	74.4	(−65.2–213.9)	40.6	(20.8–60.3)
	2009–12	10.5	(5.4–15.6)	47.9	(20.0–75.8)	68.1	(−49.4–185.6)	− 157.1	(− 280.7- -33.4)	9.2	(− 11.4–29.7)
Women	2001–04	1.3	(− 1.1–3.8)	13.6	(1.1–26.0)	57.0	(27.5–86.5)	217.6	(132.9–302.2)	37.0	(21.9–52.1)
	2005–08	2.9	(0.9–4.9)	3.2	(−4.9–11.4)	61.6	(33.4–89.8)	158.2	(78.3–238.1)	34.0	(23.9–44.0)
	2009–12	2.0	(0.2–3.8)	5.4	(−3.2–14.0)	31.9	(−3.3–67.2)	121.3	(65.3–177.3)	20.4	(10.0–30.7)
Basque Country											
Men	2001–04	15.3	(8.1–22.5)	27.7	(−0.4–55.7)	42.6	(−28.1–113.3)	338.1	(134.2–542)	69.2	(40.6–97.8)
	2005–08	15.9	(9.7–22.1)	31.3	(0.7–61.9)	64.4	(9.3–119.5)	120.2	(−22.3–262.8)	38.1	(13.8–62.5)
	2009–12	15.8	(9.9–21.7)	23.8	(−4.4–52.1)	16.5	(−47.9–80.9)	76.9	(−75.7–229.5)	26.4	(3.6–49.2)
Women	2001–04	2.5	(0–5.01)	17.7	(7.23–28.2)	31.2	(−4.8–67.2)	103.7	(−2.7–210.2)	25.1	(7.9–42.3)
	2005–08	3.2	(0.7–5.7)	2.8	(−5.0–10.6)	32.1	(5.6–58.6)	190.1	(114.3–266)	34.6	(22.6–46.6)
	2009–12	1.8	(−0.4–4.1)	4.1	(−8.3–16.4)	27.2	(5.6–48.8)	157.9	(81.5–234.3)	29.1	(16.7–41.5)

95% confidence interval in parenthesis

RII value can be interpreted as the relative inequalities in ischaemic heart disease mortality and SII as the absolute inequalities in ischaemic heart disease mortality comparing mortality rates between the two extremes of the educational spectrum (taking into account all educational levels)

If we focus on SII in Table 3, we also observe that confidence intervals overlap across periods. It is worth mentioning that SII decreases in the period of the economic crisis 2009–2012 for most groups. Comparing the two last periods, the SII in all age category for men in Barcelona decreases from 40.6 (95%CI:20.8–60.3) in 2005–2008 to a 9.2 (95%CI:-11.4–29.7) in 2009–2012, from 34.0 (95%CI:23.9–44.0) to 20.4 (95%CI:10.0–30.7) for women in Barcelona, from 38.1 (95%CI:13.8–62.5) to 26.4 (95%CI:3.6–49.2) for men and from 34.6 (95%CI:22.6–46.6) to 29.1 (95%CI:16.7–41.5) for women in Basque Country. The reduction in SII during period 2009–2012 holds for each age group except for women from 50 to 64 years in both settings.

## Discussion

We observed no significant trend in relative (RII) and absolute (SII) inequalities in IHD mortality rates according to educational levels. Regardless the stability in time trend inequalities, data show a non-significant inverted U-shape trend for RIIs and for SII for some groups under 75, except for all ages women in the Basque country and women from 50 to 64 years.

The lowest relative and absolute inequalities observed are among men. Cardiovascular disease epidemiology indicates that unhealthy risk behaviours such as smoking and a high-fat diet, among others, may account for most of the socioeconomic gradient [19, 20], potentially affecting at different degree the socioeconomic groups [20]. In Spain, the transition of smoking from upper to lower socioeconomic groups has been realized earlier for men than for women, this may explain the lower inequalities observed in men due to the oldest only, especially in Barcelona. In our study, the observed not decrease in RII and SII among women from 50 to 64 years in both regions during the economic crisis, which may be related to the unfavourable trends in tobacco consumption for women in the lower socioeconomic group [21]. Analogous dynamics of tobacco epidemics has been reported for the Basque Country [22]. Moreover, higher obesity inequalities among Basque women compared to the Spanish average may partially explain the observed increasing trend in inequalities in IHD [23]. A downward trend in fruits and vegetables intake has been reported in Spain [24] among the lowest socioeconomics groups and also in Greece [25] suggesting possible future increases in inequalities in cardiovascular mortality.

The SII decreased probably due to the improvement in access to clinical treatment and to primary care. In Spain, the extensive use of drug treatment for vascular risk factors during the last decades has been shown to be significantly associated with time decreasing rates of IHD hospitalisation [26]. The cuts on health spending practiced during the economic crisis have affected mostly outpatient prescription drugs and patients in long waiting visits time for health care, which is expected to limit the accessibility to health services more to those in the lower socioeconomic position. Certainly, these austerity policies have not been so devastating as in the Greece [27] as to reverse the reductions in inequalities in IHD trends achieved during the last decade [28]. For instance, in Norway and Finland, both countries characterized with universal health care, disadvantaged socioeconomic groups were not hit harder by pro-cyclical mortality than advantaged groups did [7, 9].

Pollution could affect many individuals, but the effects on vulnerable populations like the children, the elderly, and most disadvantaged individuals are higher [29]. Although the inverted U-shape is not statically significant, the decreasing of the absolute inequality in mortality during the last period of economic crisis may be related to a decrease in vehicle traffic. In Barcelona area, the emission of nitrogen dioxide has been shown to be associated to IHD mortality in deprived areas for men only [30]. Pollution may interact with basal worse health status among the most disadvantaged population leading to higher mortality. A decrease in traffic congestion due to the economic downturn in period 2009–2012 may have avoided some of the IHD mortality mainly among lower socioeconomic groups contributing to a lowering in inequalities in IHD trends.

Our results differ from the increasing inequalities in mortality reported for USA during 1980 and 1990s economic crisis, where individuals with low education experienced a declining health, while those with higher education level presumably had some buffer-stock savings and prospects of avoiding long-term unemployment [6]. A fact related to the meanstested provision of the public medical insurance program for Americans under age 65 years, leaving a large portion of the working poor without medical insurance. Although non-significant, our results of a inverted U-shape inequalities found in some age groups in Spain gives a weak support for this procyclical trend. In contrast, inequalities in Japanese males aged 30–59 during the economic crisis of period 1980–2010 did not increased as mortality rates among unemployed people steadily decreased for IHD perhaps because the basic properties of Japan’s social welfare system were maintained even during economic recession and mortality trends among the japanese managerial classes remained higher relative to the other occupations due to overwork and long working hours [9]. Again, in Norway and Finland characterized with universal health care, disadvantaged socioeconomic groups were not hit harder by pro-cyclical mortality than advantaged groups did [7, 8]. However in New Zealand, the socioeconomic inequalities, due to the strong restructuring of the economy, grew more than in the Nordic as a consequence RII in cardiovascular mortalities increased [31].

For Spain, our results confirms previous research reporting no changes in downward trends before and during the economic crisis for cardiovascular disease [11]. It has also been reported a non-significant increase in inequalities in cardiovascular mortality during the economic crisis [32]. Although non-significant, our results of an inverted U-shape inequalities found in some age groups gives a weak support for the procyclical trend. So that, the European Mediterranean countries most hit by the Great Recession, cardiovascular mortality appears to follow different trajectories. In Greece, the downward trend in cardiovascular mortality does not differ significantly between the pre- and post-crisis periods, although the overall decline ended in 2011–2012 [33]. In Italy, cardiovascular mortality was found to be positively associated with the unemployment rate, and showed a significant increase in 2010 [34]. In sum, our result of a stable trend in inequalities in IHD mortality is akin with the recent strand of research that stresses mild effects of economic crisis on health outcomes [35, 36].

A limitation of our study is that we cannot determine causality between the observed trends in IHD mortality inequalities and the economic crisis. Caution is needed in generalising these results to other regions, as the impact of economic recession may be different in each region.

## Conclusions

Our data confirm the existence of moderate IHD mortality inequalities, with a gradient of increasing rates through less educational level, but we did not find evidence of increasing trends in IHD socioeconomic inequalities during the economic crisis neither in relative nor in absolute terms.

## Abbreviations

CI: Confidence interval
ICD-10: International Classification of Diseases 10th
IHD: Ischaemic heart disease
OECD: Organization for Economic Co-operation and Development
RII: Relative index of inequality
SII: Slope index of inequality
